# Testosterone deficiency promotes arterial stiffening independent of sex chromosome complement

**DOI:** 10.1186/s13293-024-00624-0

**Published:** 2024-06-06

**Authors:** Anil Sakamuri, Bruna Visniauskas, Isabella Kilanowski-Doroh, Alexandra B. McNally, Ariane Imulinde, Anne Kamau, Divya Sengottaian, John McLachlan, Montserrat Anguera, Franck Mauvais-Jarvis, Sarah H. Lindsey, Benard O. Ogola

**Affiliations:** 1 Vascular Biology Center and Department of Medicine, Medical College of Georgia at Augusta University, Augusta, GA USA; 2 Department of Pharmacology, Tulane University, New Orleans, LA USA; 3 Division of Rheumatology, Department of Medicine, Perelman School of Medicine at the University of Pennsylvania, Philadelphia, PA USA; 4 Tulane Center of Excellence in Sex-Based Biology & Medicine, New Orleans, LA USA; 5 Southeast Louisiana Veterans Healthcare System Medical Center, New Orleans, LA USA; 6 Deming Department of Medicine, Section of Endocrinology and Metabolism, Tulane University, New Orleans, LA USA

**Keywords:** Sex hormones, Sex chromosomes, Vascular mechanics, Pulse wave velocity, Arterial stiffening

## Abstract

**Background:**

Sex hormones and sex chromosomes play a vital role in cardiovascular disease. Testosterone plays a crucial role in men’s health. Lower testosterone level is associated with cardiovascular and cardiometabolic diseases, including inflammation, atherosclerosis, and type 2 diabetes. Testosterone replacement is beneficial or neutral to men’s cardiovascular health. Testosterone deficiency is associated with cardiovascular events. Testosterone supplementation to hypogonadal men improves libido, increases muscle strength, and enhances mood. We hypothesized that sex chromosomes (XX and XY) interaction with testosterone plays a role in arterial stiffening.

**Methods:**

We used four core genotype male mice to understand the inherent contribution of sex hormones and sex chromosome complement in arterial stiffening. Age-matched mice were either gonadal intact or castrated at eight weeks plus an additional eight weeks to clear endogenous sex hormones. This was followed by assessing blood pressure, pulse wave velocity, echocardiography, and ex vivo passive vascular mechanics.

**Results:**

Arterial stiffening but not blood pressure was more significant in castrated than testes-intact mice independent of sex chromosome complement. Castrated mice showed a leftward shift in stress–strain curves and carotid wall thinning. Sex chromosome complement (XX) in the absence of testosterone increased collagen deposition in the aorta and Kdm6a gene expression.

**Conclusion:**

Testosterone deprivation increases arterial stiffening and vascular wall remodeling. Castration increases Col1α1 in male mice with XX sex chromosome complement. Our study shows decreased aortic contractile genes in castrated mice with XX than XY sex chromosomes.

**Graphical Abstract:**

**Figure:**
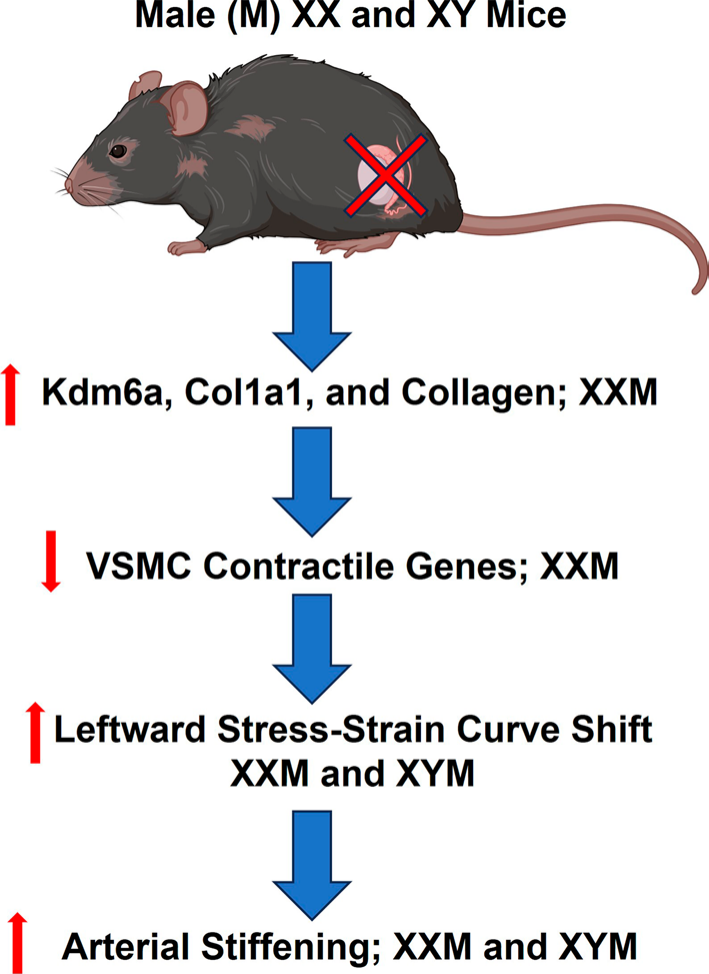

**Supplementary Information:**

The online version contains supplementary material available at https://doi.org/10.1186/s13293-024-00624-0.

## Plain Language Summary

Cardiovascular disease is the leading cause of death worldwide. Cardiovascular disease presents differently in men and women. While men develop plaque buildup in large arteries, women develop buildup in the microvessels in the heart. Arterial stiffening, which is the hardening of arteries, increases with age in both men and women. Aging, coupled with the decline in sex hormones, exacerbates cardiovascular disease in women compared to men. Men with XY sex chromosomes have higher circulating testosterone, while women with XX sex chromosomes have increased circulating estradiol. The potential benefits of sex hormone replacement therapy are shown in men and women. Indeed, testosterone replacement deficiency is associated with adverse cardiovascular outcomes in men. Whether adverse events are dependent or independent of sex hormones' interaction with sex chromosomes is unknown. This study used the four core genotype mice comprising males with either XX or XY sex chromosome complement. We show castration increases arterial stiffening and collagen deposition on the arterial wall. We also identified the escapee and smooth muscle contractile genes that may play a role in arterial stiffening. Our data suggests that testosterone deprivation mediates arterial stiffening and remodeling.

**Supplementary Information:**

The online version contains supplementary material available at https://doi.org/10.1186/s13293-024-00624-0.

## Highlights

- Castration increases pulse wave velocity independent of blood pressure in male FCG mice.
- Castration-mediated carotid artery leftward shift in stress–strain.
- Arterial remodeling, including collagen deposition and decrease in contractile genes, was more significant in mice with XX sex chromosome complement.

**Supplementary Information:**

The online version contains supplementary material available at https://doi.org/10.1186/s13293-024-00624-0.

## Background

Endogenous sex hormones, estradiol in women and testosterone in men, prevent cardiovascular disease (CVD); however, it is debatable whether the benefits are mediated by sex hormones, sex chromosomes, or their interaction [1–3]. Sexual dimorphism in mammals plays a significant role in the development of CVD, including atherosclerosis and arterial stiffening [4, 5]. Arterial plaque develops in men's large conduit blood vessels, while microvessels are affected in women who experience increased rupture during menopausal years [6]. Pulse wave velocity (PWV), an indicator of arterial stiffening, is an independent predictor of cardiovascular mortality and events [7]. Testosterone deficiency promotes higher blood pressure and arterial stiffening [8]. The Baltimore Longitudinal Study of Aging showed increased PWV and blood pressure in men compared to women, widening the sex difference gap [9]. Studies have shown that PWV either precedes or comes after the development of hypertension [10, 11]. Even so, increasing arterial stiffening is associated with cognitive decline, impairment of kidney function, and CVD in a sex-dependent and independent manner [12–14].

Sex chromosomes (XX and XY) independent of sex hormones show sex differences in females and males [15]. The male Y chromosome is inherited paternally from father to son [15]. The Y chromosome encodes the Sry gene that instructs the formation of male gonads (testes) and spermatogenic pathways [16]. The absence of testes makes cells differentiate into ovaries, indicated by the oogenesis pathway [16]. Association of the Y chromosome with hypertension is shown in the spontaneously hypertensive rat with identification of a Y-linked locus, suggesting a Y chromosome linkage to blood pressure [17, 18]. Recently, it was shown that the hematopoietic loss of the Y chromosome contributes to cardiac dysfunction [19]. Altogether, the contribution of sex chromosomes is equally significant to sex hormones in understanding and delineating sex differences in CVD.

The X chromosome comprises less than five percent of the human genome and plays a significant role in sex differences across various tissues, including the heart, aorta, and adipocytes [20–22]. While females have two X chromosomes, males have one copy [23]. For dosage compensation of X-linked genes, one of the X chromosomes in female cells is randomly selected for inactivation [24]. X chromosome inactivation (XCI) is initiated and maintained by the long non-coding RNA X inactive specific transcript (Xist), along with various heterochromatic histone modifications and DNA methylation [25]. The inactive X chromosome is epigenetically distinct from the active X in female cells [26]. Genes that escape XCI are expressed from the inactive and active X chromosomes [20]. The XCI escapee genes play a crucial role in CVD, including atherosclerosis and fibrosis in valvular interstitial cells [4, 20, 27, 28].

To understand the contribution of sex hormones, sex chromosomes, and their interaction in CVD in males and females, we used the four-core genotype (FCG) mouse model [29, 30]. In this mouse model, the Sry gene has been deleted from the Y chromosome and inserted into an autosome, resulting in (XX and XY^−^) females with ovaries and (XX and XY^−Sry^) males with testes [29, 30]. Therefore, mice can be used to test the effect of sex hormones, sex chromosomes, and their interaction. In this study, we sought to clarify whether the interaction of sex hormones and sex chromosomes promotes arterial stiffening in male mice.

## Methods

### Animals

Four core genotype (FCG) mice on C57BL/6J background were used. Studies were conducted in mice between 15–16 weeks old. Male (M) mice XY^−Sry^ were obtained from Dr. Franck Mauvais-Jarvis and were mated with female (F) breeders (XX) on C57bl/6J (RRID: IMSR_JAX:000664) background purchased from Jax Labs (ME, USA). FCG mice arise from breeding an XX female and XY^−Sry^ male mouse. Since Sry transgene is in chromosome 3, an autosome, there is independent segregation of the Y chromosome and Sry transgene during meiosis, giving rise to the four genotypes, including XXF, XXM, XYF, and XYM [30]. Mice were genotyped with the following primers: Transgene Forward Sry: 5′ AGC CCT ACA GCC ACA TGA TA 3′, Transgene Reverse Sry: 5′ GTC TTG CCT GTA TGT GAT GG 3′, Forward: 5′ CTG GAG CTC TAC AGT GAT GA 3′, Reverse: 5′ CAG TTA CCA ATC AAC ACA TCA C 3′, Internal positive control: 5′ CAA ATG TTG CTT GTC TGG TG 3′ and Internal positive control: 5′ GTC AGT CGA GTG CAC AGT TT 3′. All mice were maintained at Tulane University in a temperature-controlled vivarium under a 12-h dark and light cycle with free access to standard chow and drinking water. Animal experiments followed the Animal Research: Reporting of In Vivo Experiments guidelines [31] that Augusta and Tulane University Institutional Animal Care and Use Committee approved.

### Castration

Mice were at 8 weeks of age, followed by an additional 8 weeks to clear endogenous testosterone predominantly from the testes. All procedures followed aseptic techniques. Briefly, mice were weighed and placed under a heating pad with 3–4% isoflurane-oxygen mixture, eye cream was applied, and hair was shaved around the incision site. Alcohol (70%) and betadine were used to clean incision sites. This was followed by administration of Buprenorphine 0.1 mg/kg. Castration was performed by locating the scrotum and making a small midline cut above the bladder, followed by an excision of the testicles. Muscle tissue was sutured with absorbable suture, and the skin was stapled. Mice were left on heating pads to recover and monitored for pain and stress. Awake and alert mice were placed back in their cages, and daily post-surgical monitoring was performed for wound healing and distress.

### Tail cuff plethysmography

Blood pressure (BP) was measured non-invasively using the CODA 4-Channel Blood Pressure System (Kent Scientific, Torrington, CT) in conscious mice as previously described [32]. Three days of acclimation followed by one week of BP measurements in the morning when castrated and gonadal intact mice were 16 weeks of age. The tail temperature and platform were warmed to 30 ℃ before beginning the inflation protocol, which consisted of 10 cycles of cuff inflation to 250 mmHg followed by a 20-s deflation. Measurements without a definitive inflection point indicating the return of blood flow or with a blood flow volume of less than 30 µl were excluded. The average BP was recorded daily, and the final BP was reported as the mean of the daily averages after excluding days with ± 2 standard deviations [32, 33].

### Pulse wave velocity and echocardiography

PWV was performed as previously described by our group [32, 33]. Briefly, Vevo® 1100 ultrasound (VisualSonics, Toronto, ON) was used for cardiovascular analysis. Anesthesia was induced using a 3% isoflurane/oxygen mixture, and data was obtained under a 1.5% isoflurane/oxygen mixture. Mice were maintained in a supine position on a 37 °C heated electrocardiogram platform. Shaving cream was applied to the chest, abdomen, and around the throat and wiped with wet gauze. Intracarotid and abdominal pulse wave velocity PWV were measured. Cardiac function was assessed in short axis view in M-mode for left ventricular function.

### Passive biaxial mechanical testing

The carotid artery was dissected and cannulated onto a 500 µm needle secured with a nylon suture on a pressure myograph system in Hank’s Balanced Salt Solution, as previously described [32, 33]. Pressure was applied to the vessel's lumen, and the outer diameter was optically tracked. Biaxial phenotyping was performed as previously described [32, 33]. Pressure-diameter preconditioning was performed from 10 to 150 mmHg. The stretch ratio was assessed by dividing the loaded to unloaded axial length and used to determine the adjusted wall thickness during pressurization. Distensibility was calculated as the percent of the starting external diameter. Stress was calculated as (σ) = (P in dyns/cm^2^*D_internal_)/(2*wall thickness), while strain (ε) = D_internal_-D_10mmHg_/D_10mmHg_.

### Quantitative polymerase chain reaction

Mice aorta samples were homogenized in lysis buffer (RLT buffer, Qiagen) using a bead homogenizer. The RNA was isolated using RNeasy plus mini kit (Qiagen, cat. No. 74136). Finally, the RNA pellet was resuspended in 25 µl of Rnase-free water, and the nano spectrometer measured purity and concentrations (Implen Nano photometer, N50). cDNA synthesis was performed with superscript IV Mater mix (cat. No. 11766050). The real-time PCR was conducted using PowerTrack SYBR master mix (cat. No A46109) on QuantStudio 3 Real-Time PCR System (Applied Biosystems). The comparative cycle method (2^–(ΔΔCt)^) was applied for gene expression analysis, and the β-actin gene was used as an internal control. All the target gene primers were procured from IDT technologies; the IDT predesigned primer IDs were mentioned in the supplementary (Table 1).

### Histology

Aorta samples were fixed in 10% formalin overnight, followed by paraffin embedding. Paraffin blocks were serially cut into 5 µm sections and stained Masson’s trichrome (MTC: Blue-Collagen; Red-Muscle) or Van Gieson's stain (Black-elastin bands). Immunofluorescence staining was done using Col1a1(CST#72026), Alpha-Smooth Muscle Actin Monoclonal Antibody 1A4 (RRID: AB_557419), and DAPI (ThermoFisher, D1306). Fluorescent anti-rabbit (RRID: AB_143165) and anti-mouse (RRID: AB_2536180) Images were taken using the EVOS cell imaging system. Analysis was done using ImageJ software expressed as a percent of the total area fraction.

### Transmission electron microscopy

Testicles from XYM and XXM mice were fixed in paraformaldehyde and glutaraldehyde in 0.1 M sodium cacodylate (NaCac) buffer, pH 7.4. Postfixing was performed in osmium tetroxide in NaCac, stained in a block with uranyl acetate, dehydrated with an ethanol series, and embedded in the Epon-Araldite resin. The block was trimmed to permit proper orientation of the testicle during imaging. The diamond knife was used to cut sections on a Leica EM UC7 ultramicrotome (Leica Microsystems, Inc, Bannockburn, IL), collected on copper grids, and uranyl acetate and lead citrate were used for staining. JEM 1400 flash transmission electron microscope (JEOL USA Inc., Peabody, MA) was used for imaging with a CMOS CCD camera.

### Statistics

Data was analyzed using GraphPad Prism version 9.1 (GraphPad Software, San Diego, CA). Outliers were identified by the ROUT method (Q = 1%) [34]. 2-way ANOVA was used to compute the main effect (effect of castration and sex chromosomes), while Sidak’s multiple comparisons test was used to determine the difference between groups [35]. We used the two-stage step-up method by Benjamini, Krieger, and Yekutieli to correct for multiple comparisons, given its efficacy in controlling the false discovery rate while allowing for a more robust detection, especially to compare the effect of castration and sex chromosomes [36]. This method was used when we compared each variable at a fixed pressure. All data are presented as means ± S.E.M. All P values were not adjusted; therefore, the nominal P-values were used, and P < 0.05 was considered significant.

## Results

### Castration increases pulse wave velocity independent of blood pressure

**Fig. 1:**
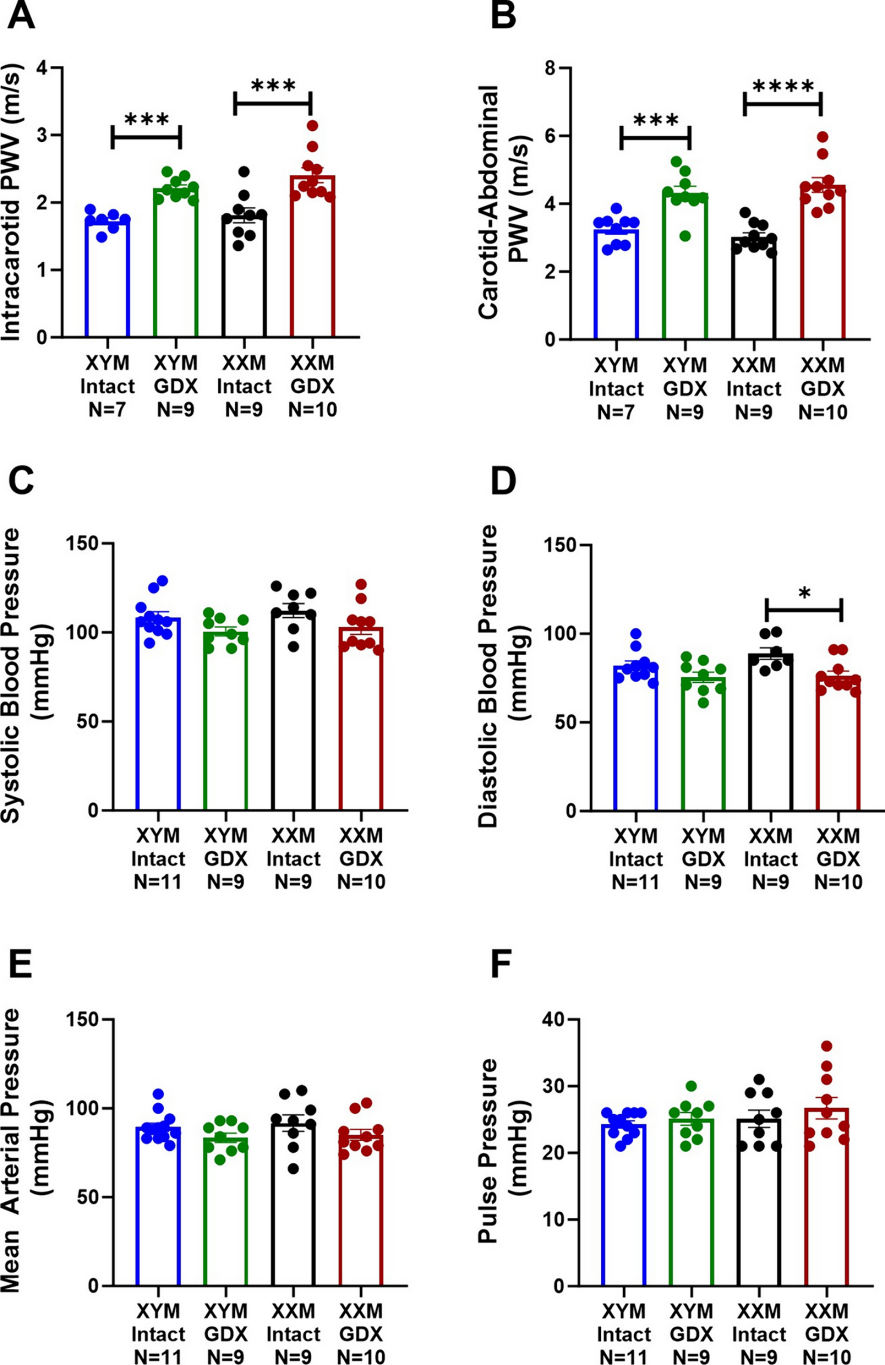
Increased arterial stiffness is not coupled with blood pressure change in gonadectomized mice. **A** Intracarotid PWV was significantly higher in gonadectomized than gonadal intact mice in XYM and XXM [(F (1, 33) = 23.9) ***P < 0.001]. Similarly, **B** Carotid-Abdominal PWV was higher in gonadectomized than gonadal intact mice in XYM and XXM [(F (1, 36) = 35.9) ***P < 0.001]. Blood pressures including **C** Systolic [(F (1, 34) = 6.1) P = 0.02] and **D** Diastolic [(F (1, 32) = 10.9) P = 0.002] were significantly different but not **E** Mean Arterial [(F (1, 36) = 3.9) P = 0.06] and **F** Pulse [(F (1, 35) = 1.1) P = 0.002]. All data sets were computed as mean fold change ± SEM, and 2-way ANOVA was used to compute the column effect, row effect, and their interaction. Post hoc Sidak’s multiple comparisons test was used. *P < 0.05, **P < 0.01, ***P < 0.001 and ****P < 0.0001 were considered significant

Intracarotid PWV was higher in castrated than testes intact mice (Fig. 1A; P < 0.0001). Post hoc comparisons indicated increased arterial stiffening in testes intact vs. gonadectomized (GDX) mice in (XYM: 1.7 ± 0.1 vs. 2.2 ± 0.1 m/s; P = 0.002) and (XXM: 1.8 ± 0.1 vs. 2.4 ± 0.1 m/s; P < 0.0001). Similarly, carotid to abdominal PWV indicated a significant castration effect (Fig. 1B; P < 0.0001). Multiple comparisons tests showed increased PWV in testes intact vs. GDX mice in (XYM: 3.2 ± 0.1 vs. 4.3 ± 0.2 m/s; P = 0.001) and (XXM: 3.0 ± 0.1 vs. 4.6 ± 0.2 m/s; P < 0.001). There was no significant main effect of castration on systolic blood pressure (Fig. 1C) in XYM (108 ± 3 vs. 100 ± 2 mmHg, t = 1.7, DF = 34; P = 0.2) and XXM (112 ± 4 vs. 103 ± 4, t = 1.8, DF = 34; P = 0.1) mice. However, diastolic blood pressure (Fig. 1D) showed significant castration effect in XXM (89 ± 3 vs. 76 ± 3, t = 3, DF = 32; P = 0.01) but not in XYM (82 ± 2 vs. 75 ± 3 mmHg, t = 1.7, DF = 32; P = 0.2) mice. Mean arterial pressure (Fig. 1E) was not significantly different in castrated versus testes intact XYM (89 ± 2 vs. 83 ± 3 mmHg, t = 1.4, DF = 36; P = 0.3) and XXM (91 ± 5 vs. 85 ± 3, t = 1.4, DF = 36; P = 0.3) mice. We also calculated the difference in systolic and diastolic pressures shown by pulse pressure (Fig. 1F) that was not significantly different in XYM (24 ± 1 vs. 25 ± 1 mmHg, t = 0.8, DF = 35; P = 0.3) and XXM (25 ± 1 vs. 27 ± 2, t = 0.5, DF = 35; P = 0.3) mice.

### Castration reduces distensibility in XXM mice at higher pressures

**Fig. 2:**
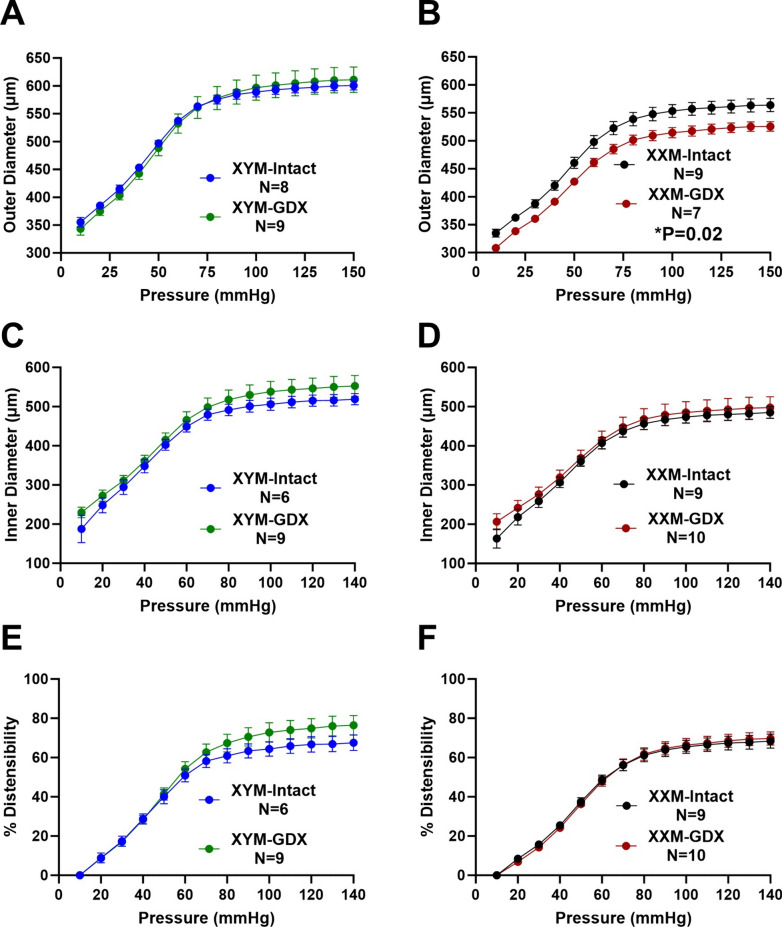
Passive pressure-diameter relationship and distensibility of carotid arteries. The pressure-outer diameter of **A** XYM was not significant (t = 0.02, df = 17; P = 0.9), and **B** XXM gonadal intact is greater than GDX (t = 4, df = 15; P = 0.0003). Pressure-inner diameters were not significantly different between castrated and intact mice in (**C**). XYM (t = 1.4, df = 13; P = 0.2) and **D** XXM (t = 0.8, df = 15; P = 0.4). **E** Distensibility was not significant in either XYM (t = 1.7, df = 15; P = 0.1) and **F** XXM mice (t = 1.2, df = 17; P = 0.8). Data presented as mean ± S.E.M. A multiple comparison test with a two-stage step-up (Benjamini, Krieger, and Yekutieli) correction was used to compare gonadal intact and GDX mice

We performed a passive pressure myograph on carotid arteries to determine whether PWV alters biaxial vascular parameters assessed ex vivo. Pressure-outer diameter relationship was not different between testes intact vs. GDX XYM (Fig. 2A: P = 0.9); however, gonadectomized XXM compared to testes-intact XXM mice showed a decrease in outer diameter (Fig. 2B; P = 0.02). The pressure-inner diameter relationship was not significantly different in castrated versus testes-intact XYM (Fig. 2C: P < 0.05) and XXM (Fig. 2D: P > 0.9) mice. To test for the elastic property of the arterial wall, we observed greater distensibility versus pressure in castrated than testes intact XYM (Fig. 2E; P = 0.1) but not in XXM (Fig. 2F; P > 0.9) mice.

### Gonadectomy decreases compliance and shifts stress–strain curves to the left

**Fig. 3:**
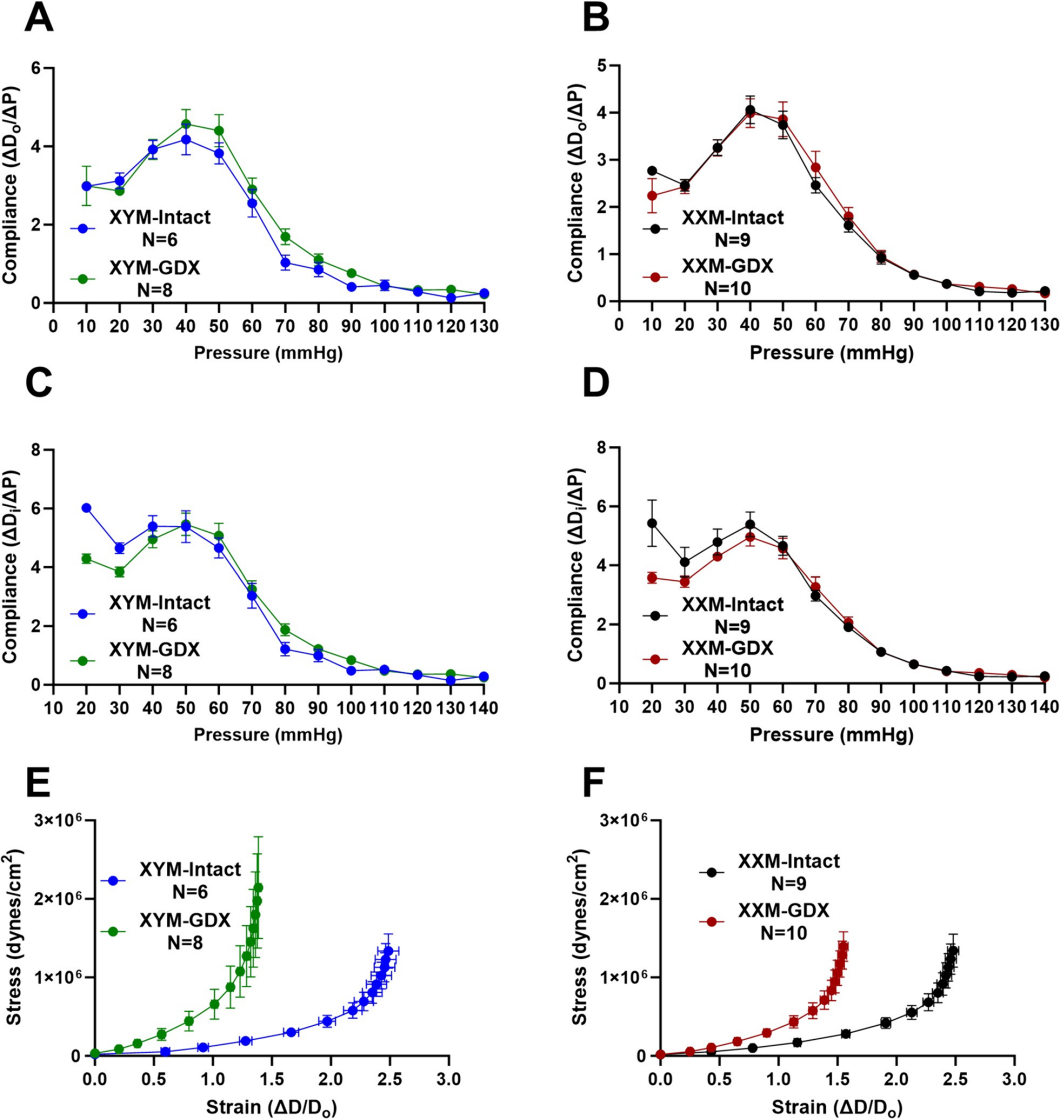
Gonadectomy decreases compliance and shifts stress–strain curves to the left. Compliance calculated from outer diameter was not different in castrated versus gonadal intact **A** XYM (t = 1.2, df = 12; P = 0.3) and **B** XXM (t = 0.3, df = 17; P = 0.8) mice. Using inner diameter dimensions also did not indicate a significant difference in compliance in (**C**). XYM (t = 0.02, df = 12; P = 0.9) and **D** XXM (t = 0.7, df = 17; P = 0.5) mice. Gonadectomy significantly shifted stress–strain curves to the left in (**E**). XYM (t = 0.07, df = 12; P < 0.05) and **F** XXM (t = 1.9, df = 17; P < 0.05) mice. Data presented as mean ± S.E.M. A multiple comparison test with a two-stage step-up (Benjamini, Krieger, and Yekutieli) correction was used to compare gonadal intact and GDX mice

Given the difference in pressure diameter and distensibility, we examined whether there were changes in compliance or a material property impacted by pressure and stiffness. Computing compliance using outer diameter changes per pressure showed no significant differences in testes intact and castrated XYM (Fig. 3A; P = 0.3) and XXM (Fig. 3B; P = 0.8) mice. Similarly, compliance calculated by inner diameter showed no significant difference in XYM (Fig. 3C; P > 0.9) and XXM (Fig. 3D; P = 0.5) in testes intact and castrated mice. Increased PWV with castration, shown in Fig. 1, prompted us to calculate the circumferential stress–strain of carotid arteries. Our data indicated increased stiffening with a leftward shift of stress–strain curves in GDX XYM (Fig. 3E; P = 0.9) and GDX XXM (Fig. 3F; P = 0.09) than testes intact mice.

### Castration-induced carotid wall thinning and aortic collagen deposition

**Fig. 4:**
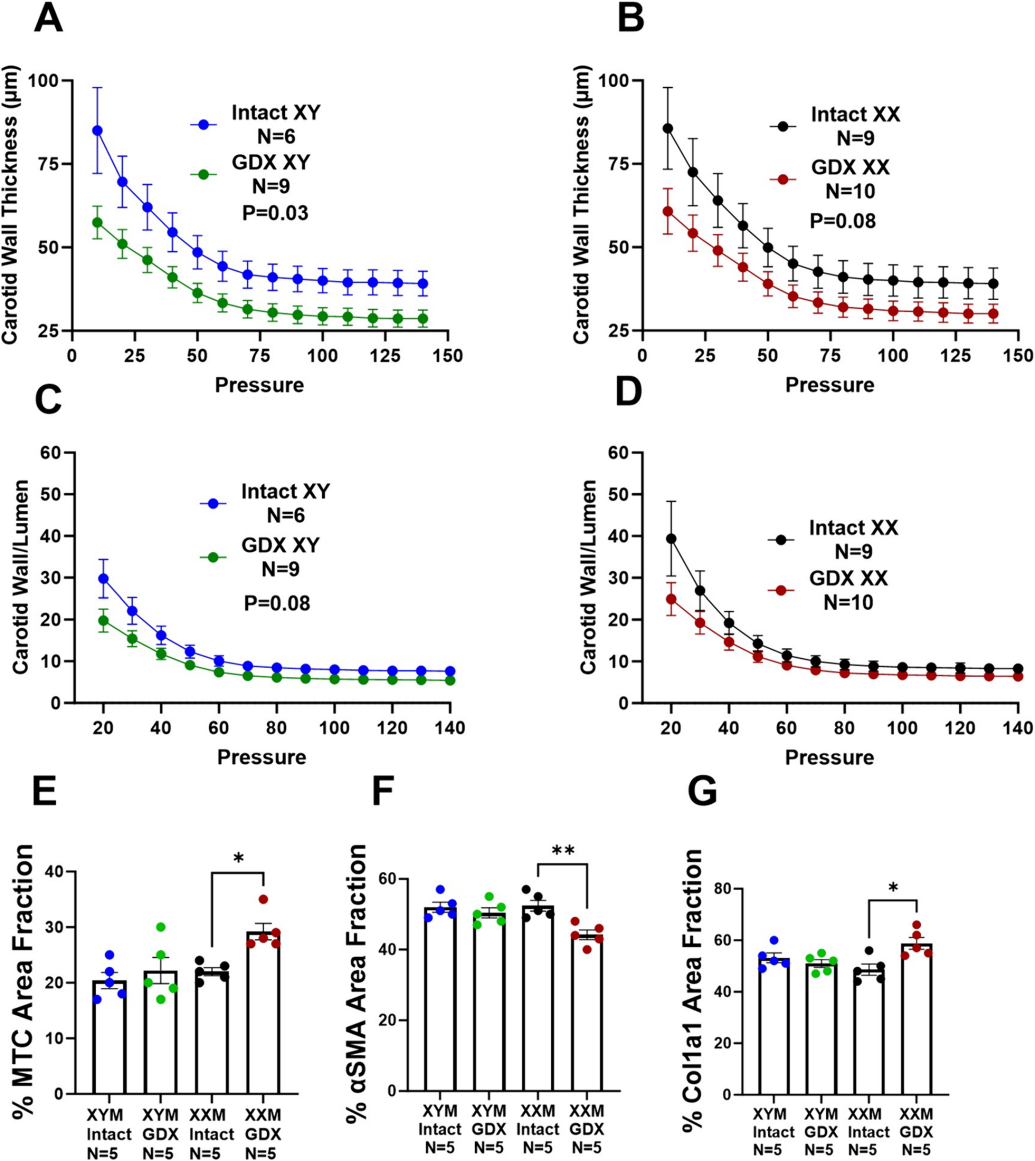

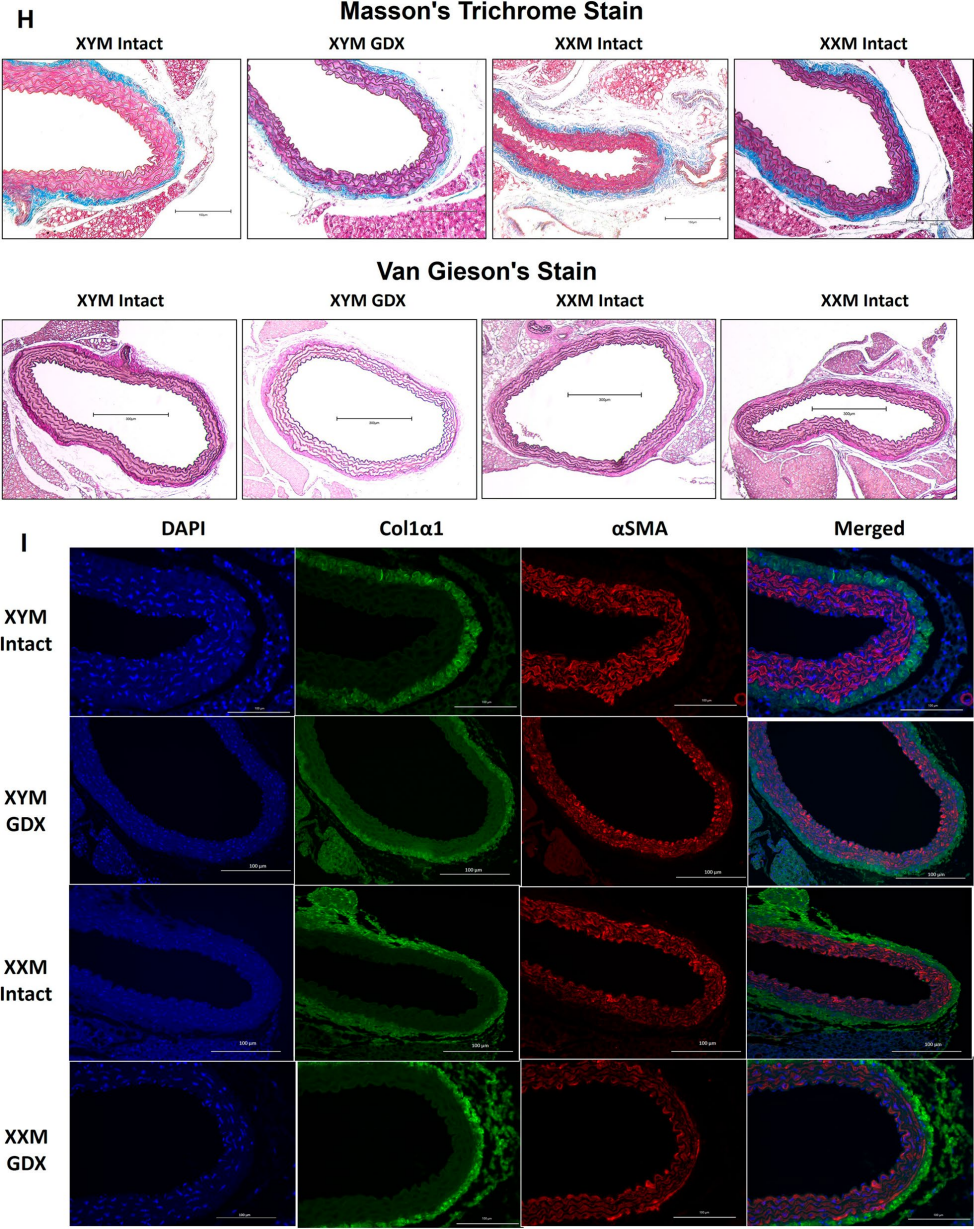
Castration-induced carotid wall thinning and aortic collagen deposition. Castration decreased carotid wall thickness in (**A**). XYM (t = 4, df = 13; P = 0.001) and **B** XXM (t = 3, df = 17; P = 0.02) mice. The carotid wall-to-lumen ratio was significantly different in (**C**). XYM (t = 3, df = 17; P = 0.009) but not in (**D**). XXM (t = 2, df = 12; P > 0.09). **E**. Gonadectomy significantly increased collagen area fraction XXM (t = 3, df = 16; P = 0.02), but not XYM (t = 0.8, df = 16; P = 0.8) mice. **F** Aortic α smooth muscle was significantly downregulated in XXM mice after castration (P = 0.005). However, **G** Col1a1 was increased in gonadectomized XX mice (P = 0.01). Representative images of Masson’s trichrome staining (**H**) and Immunofluorescence (**I**)

We assessed carotid wall thickness and wall-to-lumen ratio to determine whether testosterone deprivation mediated geometrical changes on the arterial wall. Our data uncovered the wall thinning effect due to castration versus testes intact XYM (Fig. 4A; P = 0.03) and XXM (Fig. 4B; P = 0.08) mice. Wall to lumen ratio was not significantly different in castrated versus testes intact XYM (Fig. 4C; P = 0.06) and XXM (Fig. 4D; P = 0.09) mice. Whether androgen deprivation plays a role in aortic wall remodeling is unknown; therefore, we assessed collagen deposition on the aorta using Masson’s Trichrome (Blue; Fig. 4E and H) staining and found a significant collagen increase in castrated compared to testes intact XXM (Fig. 4E: 0.2 ± 0.01 vs. 0.02; P = 0.02) but no difference in XYM mice. Representative images (Fig. 4H) show MTC staining in the top two rows and VVG staining (no elastin strand breaks; black strands) in the bottom row. To determine which collagen specifically is increasingly deposited, we performed immunofluorescence (Fig. 4I), showing smooth muscle α-actin stained red (Fig. 4F and I; P = 0.5) was decreased in castrated XXM mice. Col1a1 stained green was increased by castration (Fig. 4G and I; P < 0.05) and shown to be higher in gonadectomized than testes intact XXM mice (Fig. 4G and I; P = 0.02) but no significant difference in XYM aortic cross-sections. Representative immunofluorescence images are shown in (Fig. 4I).

### Gonadectomy downregulates contractile genes in the aorta of XX mice

**Fig. 5:**
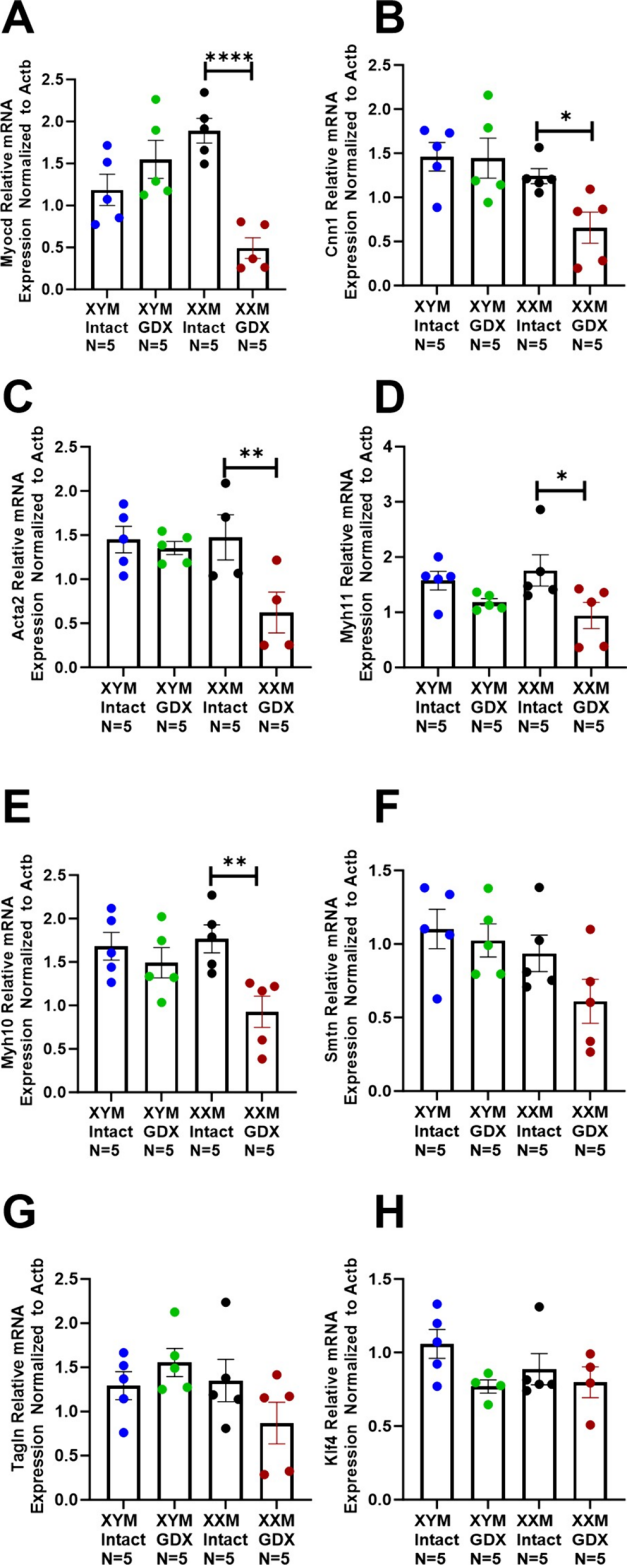
Mice with XX sex chromosome complement had a decrease in SMC contractile genes after gonadectomy. **A** Myocd (t = 6, df = 16; P < 0.0001), **B** Cnn1 (t = 2, df = 16; P = 0.03), **C** Acta2 (t = 3, df = 14; P = 0.007), **D** Myh11 (t = 3, df = 16; P = 0.01), and **E** Myh10 (t = 4, df = 16; P = 0.003), mRNA was decreased in gonadectomized XXM mice. However, there was no significant castration effect in (**F**) Smtn [(F (1, 16) = 2.4); P = 0.1], **G** Tagln [(F (1, 16) = 0.2); P = 0.6], and **H** Klf4 [(F (1, 14) = 3.9); P = 0.07]. All data sets were computed as mean fold change ± SEM, and 2-Way ANOVA was used to compute column effect, row effect, and their interaction. Uncorrected Fisher’s LSD was used for multiple comparisons *P < 0.05, **P < 0.01, and ****P < 0.0001

Collagen deposition on the aorta can modulate smooth muscle functional response; therefore, we measured contractile gene expression in testes intact and gonadectomized mice. Castration significantly decreased contractile genes in XXM but not XYM mice, including Myocardin (Fig. 5A: Myocd; P < 0.0001), Calponin (Fig. 5B: Cnn1; P = 0.03), Smooth muscle cell alpha-actin (Fig. 5C: Acta2; P = 0.006), Myosin heavy chain 11 (Fig. 5D: Myh11; P = 0.01) and (Fig. 5E: Myh10; P = 0.002). There were no significant changes in Smoothelin (Fig. 5F; Smtn), Transgelin (Fig. 5G; Tagln), and Kruppel-like factor 4 (Fig. 5H; KLF4).

### Castration increases lysine demethylase 6a gene expression

**Fig. 6:**
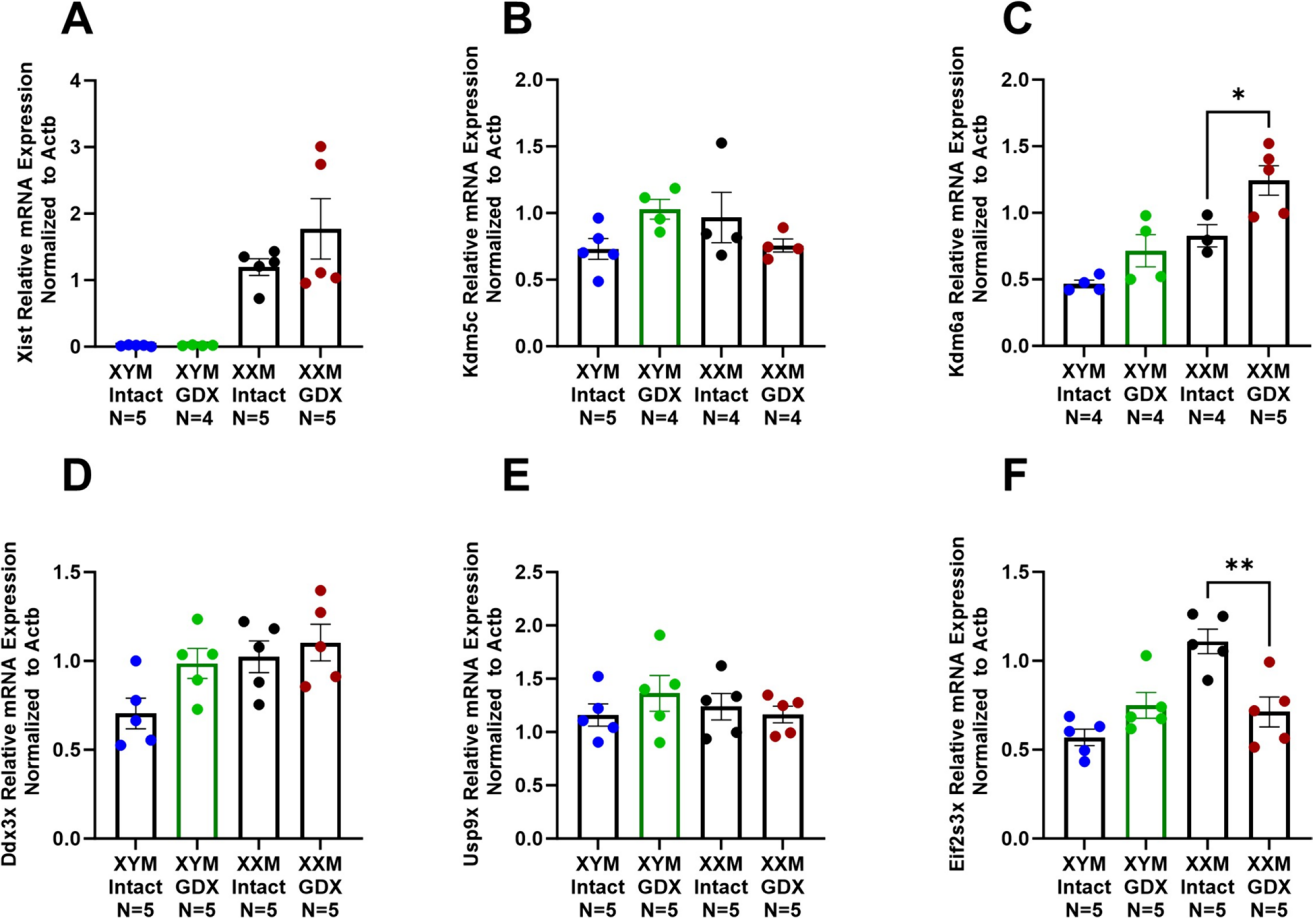
Impact of castration on X-linked gene expression in mice. **A** Distinctive Xist mRNA expression in XX than XY mice [(F (1, 15) = 34); P < 0.0001]. **B** Kdm5c expression was not impacted by gonadectomy in either XXM or XYM mice [(F (1, 13) = 0.2) P = 0.7]. **C** Kdm6a expression was significantly higher in castrated XXM mice (t = 3, df = 12; P = 0.01) but not XYM (t = 2, df = 12; P = 0.1). No significant effect of castration as indicated in (**D**) Ddx3x [(F (1, 16) = 4); P = 0.06] and **E** Usp9x [(F (1, 16) = 0.3) P = 0.6]. However, **F** Eif2s3x gene expression was decreased in gonadectomized XXM (t = 4, df = 16; P = 0.02), but not XYM (t = 2, df = 16; P = 0.2) mice. All data sets were computed as mean fold change ± SEM, and 2-Way ANOVA was used to compute column effect, row effect, and their interaction with Sidak’s multiple comparisons. *P < 0.05 and **P < 0.01

The decrease in contractile gene expression in GDX XXM but not GDX XYM compared to testes intact mice prompted us to assess X-linked escapee genes. First, we assessed Xist gene expression, distinguishing XX from XY samples. The data shows a significant (Fig. 6A; P < 0.0001) increase of Xist in GDX and testes of intact XXM but not XYM mice. Lysine demethylase 5c (Fig. 6B; Kdm5c) was not significantly different between groups. However, an increase in Kdm6a was observed in gonadectomized versus testes intact XX mice (Fig. 6C; P = 0.01). Additional escapee genes assessed, including DEAD-Box Helicase 3 X-linked (Ddx3x) and Ubiquitin Specific Peptidase 9 X-linked (Usp9x), were not significantly different between groups (Fig. 6D and E). Eukaryotic translation initiation factor 2 subunit 3, X-linked (Eif2s3x) indicated decreased expression in castrated XXM versus testes intact mice (Fig. 6F; P = 0.002).

### The anabolic effect of testosterone on tissue weights

Given the changes in vascular biomechanics, we assessed whether tissue weights were impacted by castration. Total body weight was decreased in gonadectomized XYM (Supplementary Fig. 1A: 23 ± 0.6 vs. 21 ± 0.3; t = 3, df = 31; P = 0.02) and XXM (Supplementary Fig. 1A: 25 ± 0.7 vs. 20 ± 0.5; t = 7, df = 31; P < 0.0001) compared to testes intact mice. Similarly, kidney/body weight was decreased in castrated XYM (Supplementary Fig. 1B: 6.3 ± 0.1 vs. 5.4 ± 0.2; t = 3, df = 32; P = 0.02) and XXM (Supplementary Fig. 1B: 6.8 ± 0.3 vs. 5.5 ± 0.1; t = 5, df = 32; P < 0.0001) compared to testes intact mice. Whole heart/body weight was not significantly different in castrated and testes intact XYM (Supplementary Fig. 1C: 5.3 ± 0.2 vs. 5.5 ± 0.4; t = 0.5, df = 31; P = 0.9) and XXM (Supplementary Fig. 1C: 5.6 ± 0.4 vs. 5.2 ± 0.2; t = 1, df = 31; P = 0.5) mice. However, echocardiography indicated that castration decreased in left ventricular mass in XYM and XXM compared to testes intact mice (Supplementary Table 2; 120 ± 11 vs. 98 ± 10 mg; P < 0.01 and 125 ± 6 vs. 95 ± 9 mg; P < 0.01). Testicular weight was significantly smaller in XXM compared to XYM mice (Supplementary Fig. 1D: 1.5 ± 0.2 vs. 4.6 ± 0.2; P < 0.0001). Castration significantly decreased seminal vesicle/body weight in XYM (Supplementary Fig. 1E: 9.0 ± 0.7 vs. 0.6 ± 0.1; P < 0.0001) and XXM (Supplementary Fig. 1E: 9.8 ± 0.5 vs. 0.4 ± 0.1; P < 0.0001) compared to testes intact mice.

Further evaluation of testicular morphology using a transmission electron microscope revealed structural disorganization in XXM compared to XYM mice. Leydig cells (Supplementary Fig. 2A; White arrow) were more abundant and spread out in XYM than in XXM sections. Lipid droplets (Supplementary Fig. 2B; Red arrow) were dense in XXM and scarcely distributed in the XYM section. Endoplasmic reticulum (ER) appeared to be organized and interconnected in XYM; however, XXM mice indicated swollen ER with cisternae disorganization (Supplementary Fig. 2C and D; Red asterisk). Mitochondrial morphology in XYM was elongated and numerous in XYM, but XXM showed swollen circular-like mitochondria (Supplementary Fig. 2C and D; Dollar sign).

## Discussion

For the first time, we have reported that castration induces arterial stiffening independent of sex chromosome complement without significantly changing the blood pressure of male four-core genotype (FCG) mice. We show carotid wall thinning and an overall leftward shift of stress–strain curves upon testosterone deficiency, indicating arterial stiffening. The present study shows arterial structural remodeling with increased collagen deposition, including Col1a1, without elastin strand breaks. Due to testosterone deficiency, a substantial decrease in smooth muscle contractile gene expression and an increase in the X-linked escapee gene *Kdm6a* is indicated in XXM but not XYM mice. Additionally, we show a structural disorganization of testes in XXM compared to XYM mice. The results demonstrate the role of sex chromosomes (XX and XY) and sex hormones (testosterone) in vascular biomechanics.

Aging results in a decline of sex hormones, parallel to an increase in arterial stiffening [9]. The use of androgen deprivation therapy and castration in prostate cancer increases arterial stiffening [37]. Furthermore, testosterone deficiency is associated with endothelial dysfunction, demonstrating the essential role of testosterone in vascular health [38]. A study using FCG mice on MF1 background showed no significant difference in blood pressure in XX or XY mice before Angiotensin II infusion recapitulates our findings, indicating lower but not significant systolic blood pressure after gonadectomy [39]. Besides blood pressure, PWV was higher in testosterone-deficient men without CVD, suggesting a protective role for testosterone [8]. It is shown that hypogonadal males have significantly benefited from testosterone therapy to improve libido and mood [3]. Studies have shown that gonadectomy unmasked effects of sex chromosome (XX) in hypertension, atherosclerosis, and ischemia–reperfusion injury in the heart [22, 39, 40]. We showed increased Col1a1 and Kdm6a levels in castrated XX mice, which is analogous to a study showing increased adiposity with the *Kdm5c* gene overexpression, suggesting that the lysine demethylase family of proteins plays an essential role in autosomal gene regulation [21]. However, we did not determine the mechanisms of transcriptional activation of Col1a1 by Kdm6a.

Genes that escape X-chromosome inactivation impact CVD, including atherosclerosis and fibrosis in valvular interstitial cells [4, 20, 27, 28]. We show that Kdm6a plays a role in arterial remodeling and is also implicated in autoimmunity [41]. Additionally, Kdm5c is involved in adiposity, and recently discovered escapee genes Bms and Stx regulate aortic stenosis progression [21, 27]. Kdm6a is associated with adiposity of male and female mice with XX sex chromosome complement [21, 42]. Clinically, men born with an extra X chromosome (Klinefelter syndrome) have an increased incidence of CVD that is not reversed by testosterone replacement [42, 43]. Given that men with Klinefelter syndrome have XXY sex chromosomes, lysine demethylases may be a druggable target to mitigate CVD, including arterial stiffening and metabolic disorders [43–45]. Klinefelter individuals have a Y chromosome linked to the Sry gene, unlike our mouse model (XXM), which has an autosomal Sry gene [46]. However, compared to the X, the Y chromosome encodes very few genes; even so, hematopoietic mosaic loss of the Y chromosome increases CVD risk in men [19].

Extracellular matrix, including collagen deposition and fiber arrangement on the arterial wall, has long been attributed to arterial stiffening and remodeling [32, 33]. In this study, gonadectomy increased collagen deposition in mice with XX sex chromosomes, suggesting a role for X-linked genes in driving arterial wall remodeling [22]. Our data support a role for Col1a1 in arterial stiffening and remodeling, which aligns with a study in humans and mice showing polymorphism on COL1α1 Sp1 binding site impacts arterial stiffening [47, 48]. Arterial stiffening is coupled with changes in vascular cells; we show a decrease in Myocd, a master regulator of SMC contractile phenotype changes in a similar trend to Acta2, Calponin, Myh10, and Myh11 [49]. Compelling evidence implicates SMC phenotype switching promotes aortic diseases, and our study shows how sex hormone perturbation in mice with XX sex chromosomes is impacted more [50, 51].

The arterial geometry, including wall thickness, contributes to vascular distensibility, incremental elastic modulus, and PWV [48, 52]. Our data shows that carotid walls got thinner while the aorta wall had an increase in collagen deposition, suggesting higher mechanical wall stress associated with aging and hypertension [48, 53]. Stress–strain curves indicated a leftward shift in all groups after gonadectomy recapitulating PWV assessed in vivo [11, 33]. The leftward curve shift after gonadectomy primes the vessels to respond to arterial remodeling, predisposing the vessel to impaired flow-mediated dilation, hypertension, atherosclerosis, and abdominal aortic aneurysms [22, 39, 54]. Castration decreased the body and kidney weights of XX and XY mice, suggesting that a decrease in testosterone lowered the metabolic activity and inhibited kidney growth [44]. Castrated mice fed standard chow show body weight loss, further supporting our study [55]. However, a high-fat diet significantly increases body weight in castrated mice [55].

### Perspectives and significance

While the FCG mouse is a widely considered model for investigating the role of sex hormones, sex chromosomes, and their interaction, the present study has limitations [30]. Unlike wild-type male mice, the FCG male (XYM) model has a Y chromosome independent of the Sry gene that resides in chromosome 3; therefore, we made a general assumption that the chromosomal mutation and Sry transgene location did not skew the cardiovascular phenotype [30]. An alternative approach may involve comparing male mice with a Y-chromosome mutation and a Sry transgene (XY^−Sry^) with a wild-type male mouse comprising a Y chromosome linked to Sry (XY^Sry^). Our study did not assess active vascular properties, including vasoreactivity in smaller arteries, which is shown to impact angiotensin-induced vasodilation involving the type 2 receptor [56]. The Traverse study (NCT03518034) recently showed no adverse effect of testosterone replacement in hypogonadal men, which paves the way for novel testosterone therapies in CVD [57]. Given that the presence of two X chromosomes and the Sry gene impact on CVD is rarely studied, [43] future studies can utilize the mouse XY* model that allows for the generation of mice with XXY sex chromosomes present in Klinefelter men to understand the role of sex chromosomes in vascular disease [46].

## Supplementary Information

Supplementary Material 1.

Supplementary Material 2.

Supplementary Material 3.

Supplementary Material 4.

## Data Availability

The datasets used for the current study are available from the corresponding author upon reasonable request.
