# Supplementary figures and images for: Testosterone deficiency promotes arterial stiffening independent of sex chromosome complement
Source: Biol Sex Differ. 2024 Jun 6;15:46. doi: 10.1186/s13293-024-00624-0 (PMC11155160; doi:10.1186/s13293-024-00624-0)

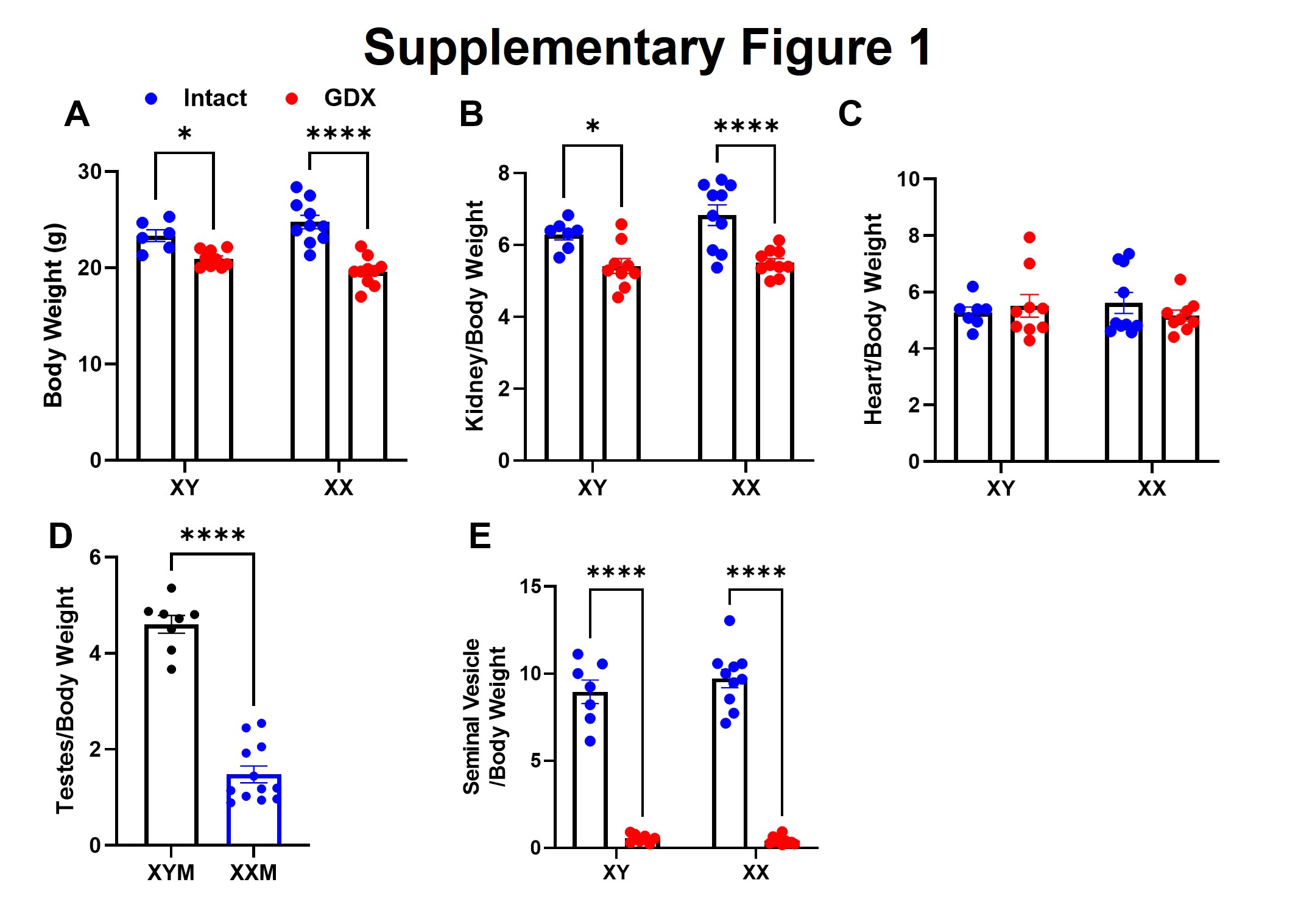

Supplement: Supplementary file 1 — Supplementary Material 1. [file 13293_2024_624_MOESM1_ESM.jpg]

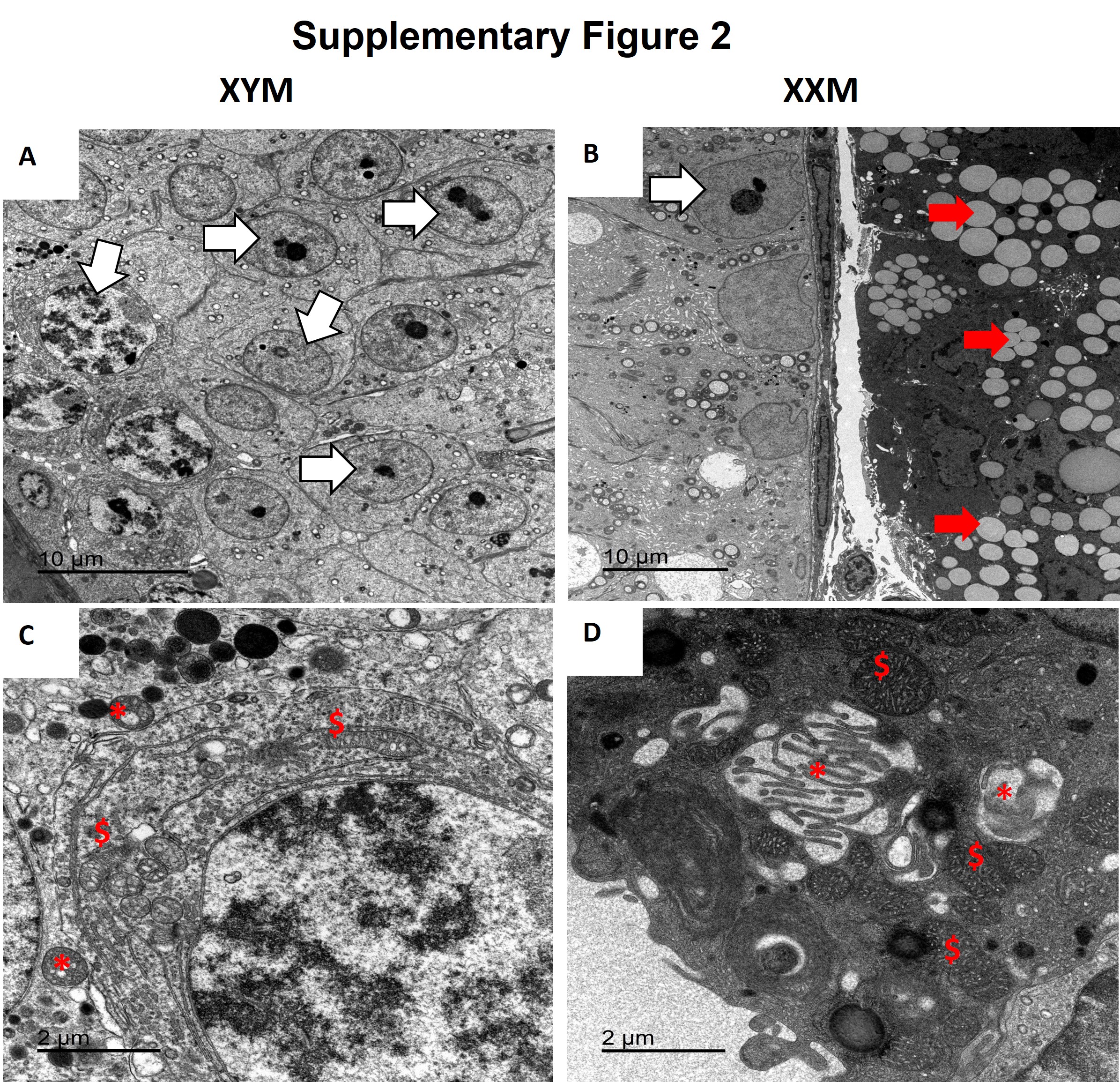

Supplement: Supplementary file 2 — Supplementary Material 2. [file 13293_2024_624_MOESM2_ESM.jpg]

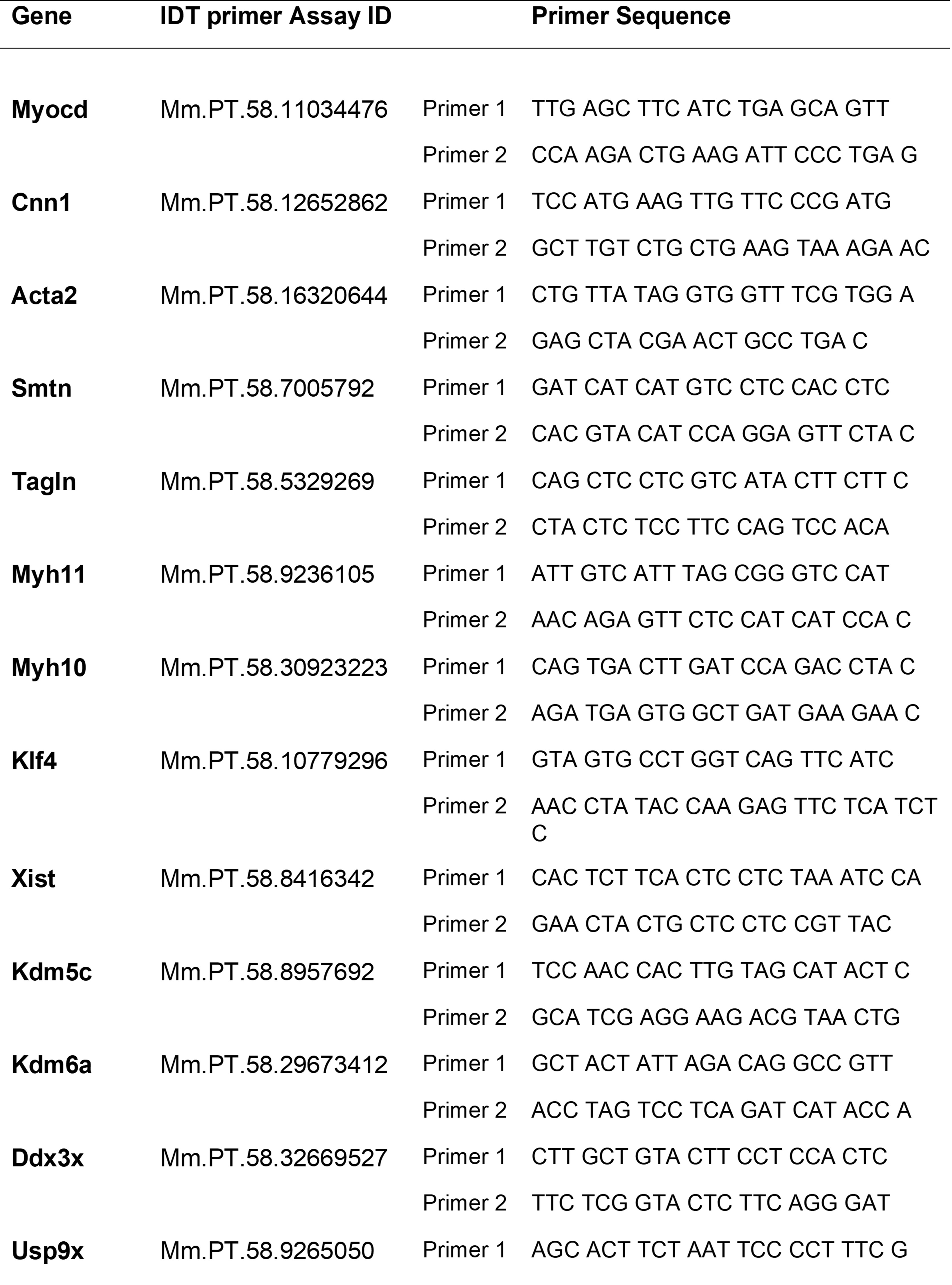

Supplement: Supplementary file 3 — Supplementary Material 3. [file 13293_2024_624_MOESM3_ESM.jpg]

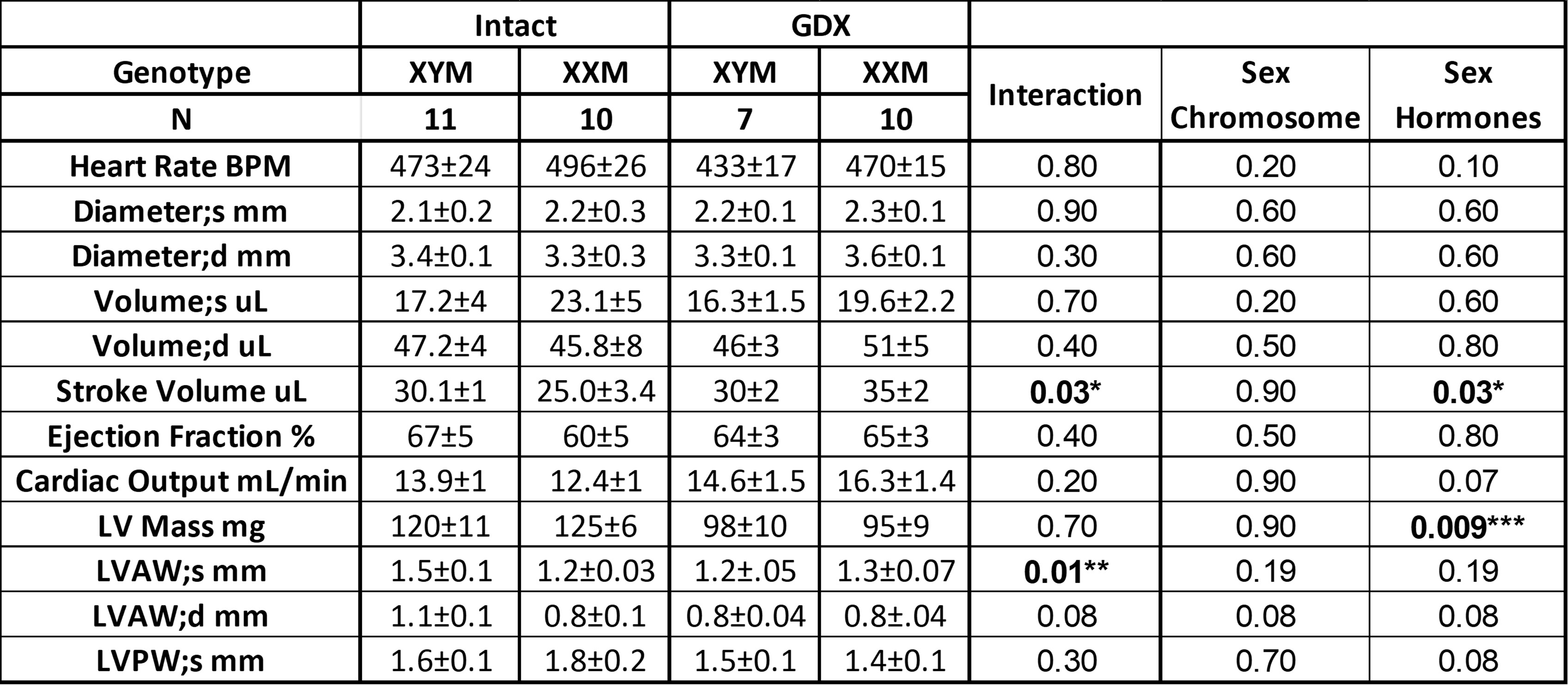

Supplement: Supplementary file 4 — Supplementary Material 4. [file 13293_2024_624_MOESM4_ESM.jpg]
